# Comparative Assessment of Antioxidant Activity and Functional Components of *Chionanthus virginicus* and *Chionanthus pubescens* from the Andean Region of Ecuador

**DOI:** 10.3390/pharmaceutics15061676

**Published:** 2023-06-08

**Authors:** Raluca A. Mihai, Iván A. Espinoza Caiza, Erly J. Melo Heras, Larisa I. Florescu, Rodica D. Catana

**Affiliations:** 1 CICTE, Department of Life Science and Agriculture, Universidad De Las Fuerzas Armadas—ESPE, Av. General Rumiñahui s/n y, Sangolquí 171103, Ecuador; 2 Department of Life Science and Agriculture, Universidad De Las Fuerzas Armadas—ESPE, Av. General Rumiñahui s/n y, Sangolquí 171103, Ecuadorejmelo@espe.edu.ec (E.J.M.H.); 3 Institute of Biology Bucharest, Romanian Academy, 296 Splaiul Independentei, 060031 Bucharest, Romania

**Keywords:** Arupo, antioxidant activity, *Chionanthus*, flavonoids, anthocyanin, inflorescence, leaves

## Abstract

The present study aims to provide information about the antioxidant capacity and secondary metabolites from different plant parts of two species that are grown in Ecuador: *Chionanthus pubescens* (the Ecuadorian national tree), and *Chionanthus virginicus* (the fringe tree—endemic to the United States of America and adapted to Ecuador’s physiographical and ecological conditions). These two species have still not been investigated for these characteristics. A comparative estimation of the antioxidant activities between the leaf, fruit, and inflorescence extracts was performed. In the quest for new medicines, the extracts were analyzed for phenolic, anthocyanin, and flavonoid content. A slight difference was observed between *C. pubescens* and *C. virginicus* flowers, the highest antioxidant activity being found in the *C. pubescens* leaf (DPPH IC_50_ = 62.8866 mg/mL, ABTS IC_50_ = 55.852 mg/mL, and FRAP IC_50_ = 2.8466 g/mL). Our results showed correlations between antioxidant activity, total phenolic content, and flavonoids. This study confirmed that the *C. pubescens* leaves and fruits from the Andean region of Ecuador represent a good source of antioxidants, especially due to the presence of a high content of phenolic compounds (homovanillic acid, 3,4 dimethoxyphenylacetic acid, vanillic acid, gallic acid, etc.) as determined by the HPLC-DAD method.

## 1. Introduction

The *Chionanthus* genus (Oleaceae family) has more than 140 species with a large distribution in the subtropic and tropic regions of America, Asia, and W. Pacific, 3 of them being in temperate regions [1].

*Chionanthus virginicus* L. (fringe tree) is a dioecious tree, endemic to the United States of America and used in traditional medicine due to its flavonoids. Considering its compounds, the root bark of the fringe tree represents the raw material of pharmaceutical industries; it is used in general for homeopathy tinctures [2] and as a woody landscape or ornamental shrub due to its fragrant white inflorescence. It is distinguished by fast growth and being free of insects and disease in the wild [3]. This species was adapted to Ecuador’s physiographical and ecological conditions [4].

*Chionanthus pubescens* Kunth (pink fringe tree, tree of love) is a tree endemic to South Ecuador to North Peru that grows in the wet tropical region. It has ornamental uses due to its pink inflorescence [5] in all the towns throughout the Andean region. In ancestral medicine, powdered tree bark was used as a purgative, and inflorescence tea was used for diuretic action. The intense red color of the fruits makes it attractive for dyeing fabrics as a natural dye and for handicrafts and ornaments; in some other places, especially in the place of origin, the wood is used to make rollers of homemade sugar mills due to its hardness [6].

In Ecuador, both species are known as “Arupo” (Arupu in Quichua, the native Ecuadorian language). The species are found in all the valleys of provinces in the Andean region from Loja to Carchi [7] at altitudes between 1.800 and 3.050 m M.a.s.l., at an average of 11–21 °C, 300–1500 mm, and 79% humidity [8].

Nowadays, there are more and more sources of pollution (environmental pollutants, radiation, unhealthy food, chemicals, etc.) that cause numerous diseases. Oxidative stress plays a key role in cellular injury, aging, cancer, neurodegenerative disease, and other disorders [9]. The reactive oxygen species lead to the depletion of antioxidants in the immune system by inducing abnormal protein formation [10]. Antioxidants (plant- and animal-based) represent compounds that prevent the oxidation of lipids and proteins [11]. Fruits, leaves, cereals, beverages, flowers, etc., are sources of antioxidants [12].

The interest in antioxidants from natural sources is constantly growing due to their capacity to prevent oxidation disorders in humans; phenolic compounds (including flavonoids) represent a major source of antioxidants that act by neutralizing free radicals [13]. Considering that both species are ornamental and, consequently, there are many leaves that can be used as a source of secondary metabolites, by demonstrating the antioxidant potential of the species *Chionanthus pubescens* compared to the species *C. virginicus* (analyzed more from the biochemical point of view), a new source of non-endangered plants with antioxidant activity could be identified, thus contributing to the conservation of the species often used for this purpose.

For this reason and the lack of information about these *Chionanthus* species, as possible natural sources of phytochemicals with antioxidant capacity, the aim of our investigation was concentrated on comparing the antioxidant activities among the leaves, fruits, and inflorescences of these two species from Ecuador.

## 2. Materials and Methods

Plant material was represented by 10 g of each fresh healthy plant part (i.e., leaves, inflorescence, and fruits) from the two *Chionanthus* species collected from the Universidad de Las Fuerzas Armadas ESPE campus located in the Andean region of Ecuador. The harvested material was placed in cooler bags and transported to the CICTE laboratory of the university for washing with distilled water and further investigations.

### 2.1. Preparation of Plant Extract

Ten-gram fresh samples (leaves, inflorescence, and fruits) from the two *Chionanthus* species were separately extracted using 20 mL ethanol (96%) and left overnight at 4 °C. The mixture was centrifugated for 15 min at 4000 rpm, the supernatant was filtrated using Whatman filter paper No. 1, and the filtered ethanolic extracts of the two species were used for antioxidant assays and quantification of bioactive compounds (flavonoids, total phenols, and anthocyanins) as well as for the high-performance liquid chromatography with diode-array detection (HPLC-DAD) analysis.

### 2.2. Antioxidant Activity Evaluation

#### 2.2.1. Ferric Reducing Antioxidant Power (FRAP) Assay

The reducing power was determined using the method described by Rajurkar and Hande (2011) [14]. The FRAP solution was realized by mixing 0.3 M acetate buffer (pH 3.6), 10 mL TPTZ in HCl (40 mM), and FeCl_3_·6H_2_O (20 mM) in a ratio of 100:10:10 at 37 °C. A mix of 3 mL FRAP reagent, 0.1 mL sample, and 0.3 mL distilled water was measured spectrophotometrically at 593 nm after 4 min of rest. The calibration curve (y = 1.0583x − 0.199; R^2^ = 0.9193) was obtained by using 6 standard solutions of ferric sulfate (Fe_2_SO_3_) in the range of 0.125–2 mmol/L.

#### 2.2.2. The Radical Scavenging Activity Assay (ABTS)

ABTS was evaluated using the methodology described by Kuskoski et al. (2005) [15]. The radical ABTS*+ was obtained through the reaction of ABTS (7 mM with H_2_O) with potassium persulfate (2.45 mM with H_2_O). The solution was left to react for 12 h. Subsequently, it was diluted in absolute ethanol until obtaining an absorbance of 0.7 ± 0.1 at 754 nm. The standard curve (y = −0.2489x + 0.7533, R^2^ = 0.9697) was obtained using Trolox standard solutions in a range of 0.039–5 mM. The inhibition capacity of a mixture of 2 mL ABTS solution and 20 µL sample was determined after 7 min of rest at room temperature in darkness. The blank was represented by the extraction solvent (ethanol) used instead of the sample.

#### 2.2.3. The Radical Scavenging Activity Assay (DPPH)

DPPH was based on the study carried out by Naspud (2018) [16]. A 2,2′-diphenyl-1-picrylhydrazyl stock solution was prepared at a 0.15 mM concentration using 96% ethanol as solvent. Subsequently, it was diluted to reach an absorbance of 0.7 ± 0.05 measured at a wavelength of 517 nm. For the sample analysis, a mixture of 2.9 mL reagent and 0.1 mL sample incubated for 30 min in the dark was used. The absorbance was read at 517 nm wavelength with a spectrophotometer. A 0.0195–0.625 mM Trolox calibration curve (y = −0.9979x + 0.7211, R^2^ = 0.9929) was used.

### 2.3. Functional Components

#### 2.3.1. The Total Phenolic Content 

TPC quantification was based on the Folin–Ciocalteau colorimetric method carried out by Thaweesang (2019) [17]. A mixture of 0.4 mL sample, 0.4 mL Folin–Ciocalteau reagent (1 N), and 2 mL distilled H_2_O was left for 5 min after adding 0.4 mL of 20% Na_2_CO_3_ and 0.8 mL distilled H2O. Subsequently, the tubes were maintained for 1 h in darkness, and their absorbances were measured at 765 nm. For the calibration curve, gallic acid was used in a 3.90625–250 mg/L range. The results were expressed as mg gallic eq/100 g sample. The curve was expressed by the equation y = 0.0112x + 0.1759 with R^2^ = 0.9794.

#### 2.3.2. The Flavonoids Quantification 

The Flavonoids Quantification was based on the methodology applied by Pekal and Pyrzynska (2014) [18]. A mixture of 1mL alcoholic extract, 1.5 mL solvent, 0.1 mL CH_3_COONa (1 M), 0.1 mL 10% AlCl_3_, and 2.3 mL distilled H_2_O was left to incubate for 40 min, in the dark. The absorbance at 435 nm was read for each sample. For the calibration curve, quercetin was used with solutions within a range of 0.023438–1.5 mg/mL, y = 1.4566x + 0.0265, R^2^ = 0.9935. The results were expressed as mg QE/g sample.

#### 2.3.3. The Anthocyanins Quantification 

The Anthocyanins Quantification was realized using the differential pH method adopted by Barragán et al. [19]. For this method, 5 g fresh samples were used in test tubes with 5 mL of two buffer systems: potassium chloride (KCL) pH 1.0 (0.025 M) and sodium acetate (CH_3_COONa) pH 4.5 (0.4 M). The content of each tube was measured using wavelengths at 520 and 700 nm, for each buffer system. The monomeric anthocyanin calculation was performed using the following equation:

$$CAT(mg\;cianidin-3–glucosid/L)=\frac{A*PM*FD*1000}{\varepsilon *1}$$

where A represents the absorbance, PM is the molecular mass of cyanidin-3-glucoside (49.2 g/mol), ε is the extinction coefficient (26,900 L/mol·cm), and 1 is the optical path of the cell (1 cm).

### 2.4. HPLC Analyses

The *Chionanthus pubescens* fruits and leaves were the samples used for polyphenol screening through HPLC-DAD analysis. High-performance liquid chromatography with diode-array detection (HPLC-DAD) analysis was carried out using an HPLC-DAD 1260, Agilent Technologies, with a C18 (150 × 4.6 mm, particle size of 5 μm) column. The samples were frozen at −80 °C and lyophilized for 1 week. An ethanol extract was made by mixing ethanol (80:20) with 1 g of sample to analyze the samples by HPLC-DAD. After that, the extract was filtered and centrifuged.

For the chromatographic analysis [20], the mobile phase consisted of a two-solvent system, used in gradient elution. A linear gradient elution program was carried out for the separation with trifluoroacetic acid 0.1% TCA (A solvent) and 5–100% acetonitrile (B) for 40 min. The gradient elution program started with 5% B and continued with 15% B at 16.5 min, 33% B at 22.5 min, 100% B at 30.5 min, and 5% B at 35 min until 40 min. The elution flow was set to 1 mL/min, and the column temperature was 5 °C. The injection volume was set to 30 μL. Diode-array detection was set to collect data in the 200 ÷ 400 nm range. Calibration curves of the HPLC-DAD method were obtained by plotting the peak area (counts) versus the concentration of the standards (μg mL^−1^). The used reference standards were chosen because they are reported to be widely represented in the plant world and have a strong antioxidant capacity. To prepare stock reference standard solutions (100 μg mL^−1^), proper amounts of reference standards were dissolved in a 30% water solution of ethanol, filtered through a 0.45 μm syringe filter, and kept at −18 °C in 2 mL vials.

Identification and quantification analyses were performed by comparison with standard spectra at each retention time. Stock reference solutions at 10, 50, 100, 200, and 400 μg/mL concentrations were used for the calibration curves.

### 2.5. Statistical Analysis

The results of our evaluations were expressed as mean ± standard deviation (SD). Non-parametric Kruskal–Wallis tests were applied for the comparison of samples by medians. The *p*-value was computed using 10,000 Monte Carlo simulations. To establish the differences between groups, multiple pairwise comparisons by Dunn’s procedure/two-tailed test of the Kruskal–Wallis test was performed.

Being 0, the white fruit sample results were removed before computations. Post hoc Dunn’s multiple pairwise tests were performed for comparisons of independent groups to identify the statistical significance of the differences.

Non-parametric Spearman correlation was applied to test the relationship between the functional compounds (TPC and TFC) and the antioxidant activity in FRAP, ABTS, and DPPH assays. The analysis was performed using XL STAT pro 15.4.03.1729 [21]. All the analyses were realized in triplicates.

## 3. Results

### 3.1. Antioxidant Activity Evaluation

The antioxidant activity of samples from both *Chionanthus* species (native and endemic) is presented in Table 1.

The FRAP assay showed that among the three types of samples, the leaves of both *Chionanthus* species show a greater free radical reduction capacity compared with inflorescence and fruit samples. Testing the variations of FRAP and TPC concentrations in the analyzed samples highlighted concentration differences between pink *Chionanthus* organs, compared to TFC. The significant result of the Kruskal–Wallis test H (4, 10) = 12.85, *p* < 0.0001, showed that there were also differences in concentrations depending on the species concerning TFC. Thus, it was noted that FRAP from the leaf differed (*p* = 0.001) from that from the inflorescence and from the fruits (*p* = 0.03). In addition, significant differences were registered between the pink leaf and the white inflorescence (*p* = 0.04) and between samples from the pink inflorescence and white leaf (*p* = 0.01).

The antioxidant capacity as determined by the ABTS method was much weaker (Kruskal–Wallis H (4, 10) = 10.23, *p* = 0.008), with significant differences remaining only between pink leaf and pink inflorescence (0.008) and white leaf and pink inflorescence (0.01) (Table 1).

The evaluation of the samples using the DPPH method supports the results of the ABTS method, the three types of samples having close values regarding the free radical inhibition capacity. Comparison of samples by Kruskal–Wallis H (4, 10) = 13.23, *p* < 0.0001, revealed significant differences by pairwise Dunn’s procedure between leaf and inflorescence of the pink *Chionanthus* (*p* = 0.01), pink leaf and pink fruit (0.001), and white leaf and pink fruit (0.013). The antioxidant activity determined by the DPPH method did not vary significantly between the leaf and the inflorescence of the white *Chionanthus*.

### 3.2. Functional compound evaluation

The total phenolic content and the flavonoids of *Chionanthus* from Ecuador are described in Table 2.

The Kruskal–Wallis test H (4, 10) = 12.3, *p* < 0.0001, highlighted significant differences regarding the total phenolic content depending on the types of samples tested. Post hoc Dunn’s multiple pairwise comparisons showed that *Chionanthus* white leaf did not present different concentration variations compared to the pink leaf. Instead, significant differences were found between the leaf and inflorescence of pink *Chionanthus* (*p* = 0.006), leaf and fruits of pink *Chionanthus* (*p* = 0.013), white leaf and pink inflorescence (*p* = 0.013), and white leaf and pink fruit (*p* = 0.02) (Table 2).

Significant differences in response were found by the Kruskal–Wallis test H (4, 10) = 13.5, *p* < 0.0001, for the species in terms of flavonoid content. Dunn’s procedure showed different features between the leaf and fruits of the pink *Chionanthus* (*p* = 0.01), white leaf and pink inflorescence (*p* = 0.001), and white leaf and pink fruit (*p* = 0.01). The white inflorescence did not present significant variations compared to the pink inflorescence (Table 2).

### 3.3. Spearman Correlation

Testing the relationships between the functional compounds (TPC and TFC) and the antioxidant activity in FRAP, ABTS, and DPPH assays in *Chionanthus virginicus* showed positive correlations for all tested elements (Table 3). Correlations were identified between TPC and DPPH (r = 1, *p* < 0.0001) and between FRAP and ABTS (r = 1, *p* < 0.0001) in the inflorescence samples. In leaf samples, FRAP was correlated with ABTS (r = 1, *p* < 0.0001) and with DPPH (r = 1, *p* < 0.0001).

In *Chionanthus pubescens* samples, the relationships of total phenolic content (TPC) and total flavonoid content (TFC) with FRAP, ABTS, and DPPH highlighted much stronger responses of TPC being positively correlated with FRAP, ABTS, and DPPH. Instead, TFC was correlated with FRAP and ABTS. DPPH proved to be the least relevant in the *Chionanthus* pink samples in TFC synthesis (Table 4). Between parts of the plants, a positive correlation of FRAP with DPPH was observed in inflorescence (r = 1, *p* < 0.0001), and a positive correlation of TFC with TPC was observed in fruits (r = 1, *p* < 0.0001), while no significant correlations were found between the tested components in leaf samples.

Anthocyanin evaluation showed a higher content of anthocyanins in the inflorescence of pink *Chionanthus* compared to that of the white one, with means of 9.3347 and 3.89084 mg cyanidin-3-glycoside/L, respectively. The fruits from pink *Chionanthus* show a lower value than the inflorescence from the pink species (Table 5).

**Figure 1:**
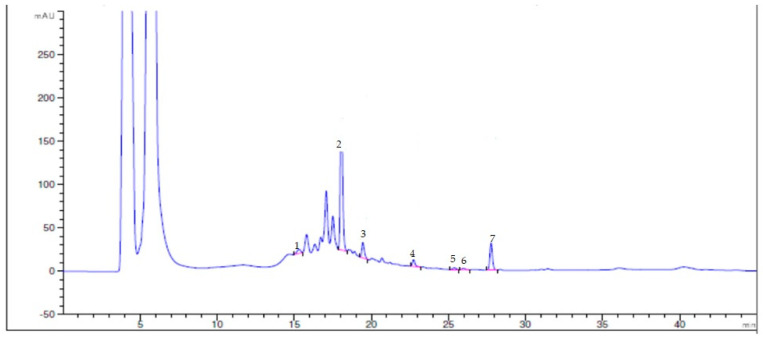
HPLC-DAD chromatogram of polyphenols from *Chionanthus pubescens* samples. 1—p-coumaric acid, 2—homovanillic acid, 3—trans-cinnamic acid, 4—3,4 dimethoxyphenylacetic acid, 5—2,5 dihydroxyphenylacetic acid, 6—vanillic acid, 7—gallic acid

Because of the higher antioxidant activity presented by fruit and leaf samples of *Chionanthus pubescens* compared to *Chionanthus virginicus* L. and because of its native origins from Ecuador and lack of investigation of this species, we verified some phenolic content by HPLC–DAD analysis. The presence of seven polyphenols in the samples was revealed, homovanillic acid being the most abundant in both fruits (75.45 mg/100 g) and leaves (58.3 mg/100 g). For both types of *Chionanthus pubescens* samples, p-coumaric acid was present in the lowest amount (<0.78 mg/100 g) (Figure 1). Trans-cinnamic acid (0.98 mg/100 g), 3,4 dimethoxyphenylacetic acid (1.78 mg/100 g), 2,5 dihydroxyphenylacetic acid (0.92 mg/100 g), vanillic acid (1.02 mg/100 g), and gallic acid (1.89 mg/100 g) could be found in higher concentrations in fruit samples than in leaves.

## 4. Discussion

Numerous studies prove the physiological functions of natural ingredients linked to the antioxidant activity of phenolic compounds [13,22,23,24] and flavonoids [25,26]. To analyze the total antioxidant capacity of natural extracts, it is necessary to apply more than one technique due to the diversity of antioxidants [27]. In this research, we applied FRAP, ABTS, and DPPH assays to determine the antioxidant activity of the two species properly (Table 1). The FRAP method hinges on the ability to neutralize the radicals through single electron transfer [28], while the others involve hydrogen donation or electron transfer [29]. In our case, the antioxidant activity of both *Chionanthus* species did not show significant differences, which underlines the potential of pink *Chionanthus* from Ecuador to be used in the pharmaceutical industry as a source of functional components with antioxidant capacity. Even though there are several studies concerning compounds with antioxidant properties of *C. virginicus* [2], there are no data concerning the analyzed properties of the aerial parts, and no data could be found for *C. pubescens*. Taking into account that the root bark (the essential part of plant survival) is the raw material for the pharmacology industry, studies concerning the antioxidant activity of other plant parts are imperatively necessary. With this aim, we investigated leaves, inflorescences, and fruits from both pink and white *Chionanthus* for their functional compounds (total phenolic content and flavonoid content) and for their antioxidant activity.

Published data with different solvents and concentrations make it difficult to compare antioxidant activity. In 2009, Gülçin et al. determined the antioxidant activity of two isolated metabolites (ligustroside and oleuropein) in root bark extracts (20 µg/mL) of *C. virginicus*, obtaining better IC_50_ values in DPPH (32.2 and 11.12 µg/mL) and ABTS (51.8 and 97.2 µg/mL) than in Trolox (62.50 and 5.38 µg/mL) [2]. In the absence of precedents in the same species, we compared the values obtained with another representative member of the *Oleaceae* family, the olive plant (*Olea europaea* L.), an economically important species with antioxidant activity. Cheurfa et al. (2019) reported an IC_50_ of 69.15 mg/mL (DPPH) for ethanolic extracts of *O. europea* leaves [30]. Duarte et al. (2012) reported an IC_50_ of 44.08 mg/mL (DPPH) in ethanolic extracts of aerial parts (wood, barks, leaves) of the same plant [31]. These data suggest a representative antioxidant activity in our analyzed samples (Table 1), mainly in leaves of pink and white *Chionanthus* (62.8866 and 66.3938 mg/mL, respectively). As both species are ornamental, the leaves may represent a valuable source of antioxidants, not a waste. In addition, leaves are considered to be very useful in the extractive process.

Carotenoids and flavonoids are the two classes of pigments responsible for inflorescence colors [32]. Sun et al. (2105) [33] describe flavonoids in *C. retusus* inflorescence. Anthocyanins, part of flavonoids, are the metabolites responsible for the reddish color of leaves, inflorescence, and fruits of different species [34], with significant antioxidant activity [35,36].

Our data present lower values than those of the other authors who studied different species from the *Chionanthus* genus. Lee et al. (2019b) [26] determined a yield of 119.1 ± 2.7 mg GA/g DW of total flavonoids and 125.4 ± 3.3 mg GA/g DW of phenolic content in *C. retusus* leaves. In the case of Chionathus pubescens, there is scarce information, and information is only available about the phytochemical composition of the fruits through qualitative phytochemical screening showing the presence of phenolic components at the fruit level [37].

The most abundant polyphenol found in our samples was homovanillic acid (HVA), a dopamine metabolite. In plants, dopamine enhances the tolerance against various environmental stresses (drought, salt stress, etc.) or biological stressors such as nutrient deficiency [38,39,40]. Our results concerning the homovanillic acid are similar to those obtained in black olives [41]. In olive leaves, HVA exhibits high antioxidative activity against lipid peroxidation induced by oxygen-centered radicals [40]. Its inhibition effect on ROS production by protecting L-6 myoblasts from cytotoxicity was demonstrated [42] by Patil et al. (2010) [43], underlining its antioxidant and antiradical activity.

Vanillic acid is an oxidized form of vanillin produced during the conversion of vanillin to ferulic acid. The highest quantity of vanillic acid in plants has been found in the roots of *Angelica sinensis*. Vanillic acid, used as a flavoring agent, is an important compound in the management of immune or inflammatory responses [44].

Gallic acid is widely spread in plants [45] and has numerous medicinal uses as a strong antioxidant in oxidative stress, being an important alternative in dietary supplements [46].

Another compound found in a significant quantity was 3,4-dimethoxyphenylacetic acid. This is an aromatic acid with antimicrobial properties, and it is used as a food additive for the preservation of meat and poultry; it inhibits cellular physiology [47].

## 5. Conclusions

The different parts of the two *Chionanthus* species grown in Ecuador have been investigated in this study, and it has been revealed that the ethanolic extracts of leaves from both species investigated showed a pronounced ability to scavenge ABTS cation radicals and antioxidant activity, as measured by the FRAP and DPPH methods. The potent antioxidative activity was associated with the high content of total polyphenols and flavonoids in the tested extracts. The antioxidant potentials of these two species were demonstrated for the first time in this present work; hence, the ethanolic extracts from different plant parts of these species may be used in pharmaceutical industries, as supplements with health potentials. These possible future applications are based on the determination of the content of some phenolic compounds (homovanillic acid, 3,4 dimethoxyphenylacetic acid, vanillic acid, and gallic acid) being very similar to that of olive oil or olive fruits. These data provide the basis for further studies investigating the usefulness of these extracts in the management and treatment of various diseases.

## Figures and Tables

**Table 1:** The antioxidant capacity of the samples from pink and white *Chionanthus*.

Sample	DPPH %RSA at 0.1 g/mL	DPPH IC_50_ (mg/mL)	DPPH R^2^	ABTS %RSA at 0.1 g/mL	ABTS IC_50_ (mg/mL)	ABTS R^2^	FRAP Pot. Red. Fe^2+^ (mM)	FRAP IC_50_ (g/mL)	FRAP R^2^
pink leaf	73.1457	62.8866	0.9127	88.7318	55.852	0.9997	1.8024	2.8466	0.9931
white leaf	71.8797	66.3938	0.9691	87.0970	55.9809	0.9994	1.8053	2.8414	0.9934
pink inflorescence	68.1114	73.3836	0.9996	83.6500	66.9785	0.9831	1.8078	2.9483	0.9976
white inflorescence	69.8689	83.4789	0.9998	76.5798	61.1557	0.9908	1.8100	2.9500	0.9977
pink fruit	65.6538	90.7797	0.9995	86.8316	58.6658	0.9900	1.8008	2.9337	0.9864
white fruit	64.4772	94.5021	0.9815	83.0756	59.9252	0.9990	1.7908	2.8769	0.9923
Trolox	*	0.08182	0.9955	*	0.3602	0.9697	*	0.0197	0.9992

Note: %RSA = radical scavenging activity, * different concentration. The IC_50_ values obtained were compared with a Trolox standard (IC_50_ = 1.4391).

**Table 2:** The total phenolic content and the flavonoids of *Chionanthus* from Ecuador.

Samples		TPC (mg GAE/g FW)	TFC (mg QE/g FW)
leaf	pink	28.4594 ± 0.4654	60.9502 ± 2.3235
	white	24.6031 ± 0.4713	66.9001 ± 2.6568
inflorescence	pink	11.2801 ± 0.5744	11.7671 ± 0.3171
	white	14.7404 ± 0.2159	17.1495 ± 1.1993
fruit	pink	11.4634 ± 0.2314	13.8176 ± 0.5419
	white	10.6721 ± 0.8806	14.0922 ± 1.3432

**Table 3:** Correlation matrix (Spearman) of the functional compounds (TPC and TFC) with the antioxidant activity in FRAP, ABTS, and DPPH assays in *Chionanthus virginicus*.

Variables	TPC	TFC	FRAP	ABTS	DPPH
TPC	**x**	0.00	0.00	0.01	0.00
TFC	**0.88**	**x**	0.00	0.00	0.00
FRAP	**0.90**	**0.93**	**x**	<0.0001	0.00
ABTS	**0.81**	**0.90**	**0.98**	**x**	0.00
DPPH	**0.95**	**0.93**	**0.95**	**0.90**	**x**

Values in bold are different from 0 with a significance level of alpha = 0.05 (the coefficient of determination r is shown in the white half; *p* is shown on the gray half of the table).

**Table 4:** The Spearman correlation between the functional compounds (TPC and TFC) and the antioxidant activity in FRAP, ABTS, and DPPH assays in *Chionanthus pubescens*.

Variables	TPC	TFC	FRAP	ABTS	DPPH
TPC		**0.008**	**0.017**	**0.014**	**0.043**
TFC	**0.828**		**0.001**	**0.002**	0.194
FRAP	**0.778**	**0.941**		**0.006**	0.194
ABTS	**0.800**	**0.904**	**0.854**		0.291
DPPH	**0.700**	0.477	0.477	0.400	

Values in bold are different from 0 with a significance level of alpha = 0.05 (the coefficient of determination r is shown in the white half; *p* is shown on the gray half of the table).

**Table 5:** Anthocyanin evaluation in pink and white *Chionanthus* tree samples.

Samples	Abs	CAT (mg cyanidin-3-glucoside/L)
pink inflorescence	0.559	9.33467658
white inflorescence	0.233	3.890840149
pink fruit	0.319	5.331118959

## Data Availability

Not applicable.
